# Genetic Clonality as the Hallmark Driving Evolution of Non-Small Cell Lung Cancer

**DOI:** 10.3390/cancers14071813

**Published:** 2022-04-02

**Authors:** Marcin Nicoś, Paweł Krawczyk

**Affiliations:** Department of Pneumonology, Oncology and Allergology, Medical University of Lublin, 20-954 Lublin, Poland; krapa@poczta.onet.pl

**Keywords:** NSCLC, tumor evolution, driver mutations, clonal/subclonal alterations, metastases

## Abstract

**Simple Summary:**

Limited knowledge about NSCLC evolution has affected therapeutic strategies for many decades. The application of NGS-based techniques to studies on ITH has provided genetic insight into the contribution of clonality primary seeding, as well as to distant dissemination. To date, multiregional ITH affects accurate diagnosis and treatment decisions and is considered the main hallmark of anticancer therapy failure. Understanding the evolutionary trajectories that drive the metastatic process is critical for improving treatment strategies for this deadly condition. In this review, we discuss how the clonality of genetic alterations influence the seeding of primary and metastatic lesions of NSCLC, highlighting that wide genetic analyses may reveal the phylogenetic lineages of NSCLC evolution.

**Abstract:**

Data indicate that many driver alterations from the primary tumor of non-small cell lung cancer (NSCLC) are predominantly shared across all metastases; however, disseminating cells may also acquire a new genetic landscape across their journey. By comparing the constituent subclonal mutations between pairs of primary and metastatic samples, it is possible to derive the ancestral relationships between tumor clones, rather than between tumor samples. Current treatment strategies mostly rely on the theory that metastases are genetically similar to the primary lesions from which they arise. However, intratumor heterogeneity (ITH) affects accurate diagnosis and treatment decisions and it is considered the main hallmark of anticancer therapy failure. Understanding the genetic changes that drive the metastatic process is critical for improving the treatment strategies of this deadly condition. Application of next generation sequencing (NGS) techniques has already created knowledge about tumorigenesis and cancer evolution; however, further NGS implementation may also allow to reconstruct phylogenetic clonal lineages and clonal expansion. In this review, we discuss how the clonality of genetic alterations influence the seeding of primary and metastatic lesions of NSCLC. We highlight that wide genetic analyses may reveal the phylogenetic trajectories of NSCLC evolution, and may pave the way to better management of follow-up and treatment.

## 1. Introduction

The Darwinian theory of variation, heredity and selection had provided a basic evolutionary framework that has been further adopted to develop models of tumor evolution [1,2]. Based on this, today we postulate that cancer may arise from normal cells as a consequence of somatic or germline genetic alterations that represent a key hallmark related to the tumorigenesis [3]. The genetic disorders may affect the cell’s survival and proliferations as “drivers” of tumorigenesis, or stay biologically neutral as “passengers” of the evolutionary processes [4]. Moreover, the alterations may be limited only to a subset of cancer cells, as clonal changes that are private within a single taxon, or subclonally distributed in all cancer cells sharing abnormalities between the trunks of the phylogenetic tree [5,6,7]. However, even a homogenous tumor with the clonal origin may accumulate additional driver alterations in the evolutionary lineage that lead to coexistence of genetically and phenotypically distinct subclones within a tumor, which is defined as intratumor heterogeneity (ITH). This provides us with the definition of clonality that in oncology refers to a uniform population of malignant cells that may be clonal or subclonal [1,2,5,8]. In this way, the genetic chaos acts as fuel for neoplastic evolution that, in consequence, leads to development of metastases [3,9,10,11]. There are many controversies about the divergence in genetic background between primary and metastatic lesions, as well as whether the primary tumor cells innately contain the capability to metastasize, or whether they acquire it throughout evolution [10,12,13,14]. Traditionally, metastatic dissemination is considered as the end-product of cancer evolution; however, clinical follow-ups indicate that cancer spread may occur at both early and late stages of the evolution [13,15]. Moreover, there is evidence that the cancer dissemination may be a consequence of clonal and subclonal seeding patterns [13,14].

The seeding of metastases takes place according to two hypothetical scenarios. In the first scenario, a new site is colonized by a single founding cell that expands by division to a detectable metastasis. In the second scenario, a continuous influx of cancer cells is responsible for the seeding of the metastatic niche [16,17]. According to the first scenario, primary tumor and metastasis share only the alterations present in the founding cell. In contrast, according to the second scenario, primary tumors and corresponding metastases share, on average, the same genetic diversity [10,15]. The distinct genotype between primary and metastatic lesions is believed to affect the response of metastases to anti-cancer therapies, and ultimately explain the failure of most therapeutics in metastatic patients [7,14]. In non-small cell lung cancer (NSCLC), at the moment of diagnosis, distant metastases are present in approximately 30–40% of patients, which results in poor prognosis. Moreover, locally advanced disease is diagnosed in approximately 50–75% of patients [18,19]. Understanding the genetic changes that drive the metastatic process is critical for improving the treatment of this deadly condition [11,13]. Application of a next generation sequencing (NGS) technique has already helped reveal the history of tumorigenesis and evolution [20,21,22]; however, further implementation of NGS and computational tools may allow reconstruction of phylogenetic clonal lineages and clonal expansion [4,23,24].

Currently, the clinical approaches in NSCLC do not consider the aspects of clonal- and subclonal heterogeneity, and mostly rely on the theory that metastases are genetically similar to the primary lesions from which they arise. Therefore, in this review, we discuss how the clonality of genetic alterations influence the seeding of primary and metastatic lesions of NSCLC. We highlight that wide genetic analyses may reveal the phylogenetic trajectories of NSCLC evolution that may pave the way to better management of follow-up and treatment. However, in this review we do not discuss how plasma gene mutations effect the process of NSCLC tumor evolution. Although understanding of this phenomenon in the context of clonal heterogeneity and tumor evolution is important, we plan to elaborate this topic in another review where we will discuss the clinical utility of liquid biopsy for circulating tumor cells analysis by novel methodology with single-cell resolution.

## 2. Clonality of Genetic Alterations

Tumorigenesis is a multi-step process involving genomic instability at both mutational and chromosomal levels [4,11,25]. Determination if the cancer drivers occur early or late in the evolutionary lineage may indicate their involvement in tumorigenesis or formation of metastases [10,13,25]. Moreover, the knowledge about driver clonality may be informative for therapeutic choices [3]. For instance, if alterations are subclonally present in only a subset of cells, potential treatment efficacy is significantly reduced [25]. It is presumed that pivotal driver alterations are present at the early stages of tumorigenesis and they trigger linearly clonal expansion that may be specific for histologic subtype [3,25,26]. Such trunk alterations are likely ubiquitous events distributed homogenously at all sites of disease; however, ubiquitous alterations alone may not be sufficient to induce formation of metastasis [3,4,27,28]. Indeed, primary tumors of NSCLC, in order to develop the metastases, need to acquire late somatic alterations that may be spatially separated between regions of the same tumor or its metastatic sites [2,27,28,29]. In this context, the alteration dominating in the trunk is the marker of clonality related to the primary and its metastatic lesions [27]. However, the high percentage of ubiquitous mutations implies the importance of targeting truncal alterations in the phylogenetic tree in order to prevent dissemination [30,31]. Moreover, the clonality may be also expressed as a ratio of the number of early mutations to the number of late mutations, which is defined as the genome doubling [25].

Literature data indicate that various subtypes of primary tumors of NSCLC are associated with exposure to environmental factors [32]. For instance, tobacco carcinogens are responsible for development of almost all LUSC (lung squamous carcinoma) and the majority of LUAD (lung adenocarcinoma), while harmful substances in air pollution are considered the initial factors for non-smoking LUAD [32,33,34]. A comprehensive analysis of the NSCLC genetic landscape reveals that it harbours predominantly clonal alterations, which occur before genome doubling, suggesting their involvement in tumor initiation [25,35,36,37,38]. Moreover, the burden of early clonal alterations correlates with the COSMIC (Catalogue of Somatic Mutations in Cancer) smoking mutational signature and a common pattern of early clonal genome doubling, followed by extensive subclonal diversification [25]. However, there is a trend that LUSC carries significantly more clonal disorders than LUAD, which may result from the later genome doubling in LUSC [25,36,37,38]. In contrast, the genome doubling LUAD is associated with the frequency of both subclonal mutations and CNAs. Moreover, mirrored subclonal LUAD allelic imbalance is significantly enriched in genome-doubled tumors. On the other hand the evidence suggests that genome doubling events are associated with the propagation of subclonal chromosomal instability by cancer cells and may predict a poor prognosis [25]. Surprisingly, smoking habits and smoking mutational signature were not correlated in LUSC, while LUAD former smokers carry late clonal mutations related with the smoking mutational signature, which might suggests a long period of tumor latency in LUAD evolution before clinical presentation [25,33,37,38]

Furthermore, in LUADs, significantly higher clonal and subclonal mutational burdens were observed in smokers than in never smokers [25]. On the other hand, the smoking signature in NSCLC was negatively associated with the risk of metastasis [3,28,39]. In contrast, the APOBEC (apolipoprotein B mRNA editing enzyme, catalytic polypeptide-like) mutational signature, which is one of the most prominent COSMIC mutation signature in neoplasms due to it affecting the tumor immune-escape, is enriched later in NSCLC than in other solid tumors [40]. The APOBEC signature in NSCLC evolution is commonly associated with subclonal mutation burden [25,28], as well with higher risk of metastasis [39].

Chromosomal instability may also be an initiator of tumorigenesis, whereby differently altered alleles may evolve in parallel, having various impacts on evolution and genetic heterogeneity [11,41]. It was estimated that around 13% of alterations may be subclonal through selective loss of genomic segments carrying clonal alterations [41,42]. It was shown that clonal and subclonal copy number alterations (CNAs) of tumor suppressors are ubiquitous across all tumor types and tend to occur as structured events, which potentially allows the continuous optimization of cellular fitness throughout tumour evolution [1,2,11,41,43]. Large ITH for CNAs in NSCLC was associated with an increased risk of recurrence or death [25]. In LUSC, both clonal and subclonal, CNAs correlated with increased cell cycle gene expression [37], while in LUAD tumor stage and Ki67 overexpression were positively associated with the proportion of subclonal CNAs [25].

There is evidence that some targetable driver alterations are clonal and appear early in tumorigenesis, while others are subclonal and tumors acquire them later during evolution [25,28]. In particular, mutations in the PI3K-AKT-mTOR pathway were found to harbour a higher proportion of subclonal mutations compared to genes associated with the RAS-MAPK-ERK pathway [3]. Mutations in the *TP53* gene appear clonal in all subtypes of NSCLC [9,28], while *KRAS* and *EGFR* genes mutations are exclusively clonal in smoking and non-smoking related LUADs, respectively [31,33,35]. A summary of clonality characteristics of commonly altered genes in literature data in primary tumors of NSCLC is presented in Table 1.

In the context of a single gene analysis, a clonal relationship cannot be proof for lineage between compared tumors [43]. Moreover, the subclonal driver mutations can give an illusion of clonality due to sampling bias [9,25]. A TRACERx study (TRAcking non-small cell lung Cancer Evolution through therapy [Rx]) has proven that 76% of subclonal mutations identified through multi-regional sequencing would appear clonal if only a single site was biopsied [25]. This observation confirms that most regions of NSCLC harbour subclones from only a single branch of the phylogenetic tree [6,13,29,44]. Moreover, due to a high degree of ITH in NSCLC, the resistant clones may already be present at the beginning of treatment or may develop later during therapy [30,45,46,47]. Single-region sampling does not provide an adequate information about the clonality of alterations in a tumor [25,42]. However, it may be sufficient to identify the majority of known trunk alterations, which include most drivers [42]. Moreover, it is clinically enough to evaluate one biopsied region of the primary or metastatic tumor, since targeting the clonal mutations is more likely to succeed compared with targeting the subclonal mutations [31,42,44,48]. However, more driver alterations may be identified by multiregional sampling [25,29,49,50]; therefore this may be used to predict which region of the tumor will be more involved in resistance or further evolution [42,49]. It is also possible that larger multiregional series may identify truncal genes in the phylogenetic tree, which may be effectively targeted in order to suppress NSCLC dissemination.

## 3. The Cell of Origin Theory

Various theories exist for the phenomenon of oncogenesis [1]. It is likely that cancer development starts from cancer stem cells (CSCs) that have the ability for hierarchical differentiation through self-renewal and asymmetrical division. In this way two different populations of ancestral cells may give rise to diverse cellular colonies that further accumulate genetic alterations and undergo selection separately [12,36]. This proves that ITH arises early in tumorigenesis [9,12,45]. Moreover, it was reported that cells may spontaneously shift between CSC-like and non-CSC-like states throughout tumor evolution under the influence of oncogenes and microenvironmental factors [12,51,52]. On the other hand, several studies have shown that CSCs are resistant to many commonly used therapies [52], thereby contributing to metastases [12,51,53,54]. However, in the CSC theory, only a small population of cells within the primary tumor is able to drive tumor dissemination [52,53]; thus, targeting them would improve the treatment outcome [7,55].

There are many studies that aim to extract and characterize the NSCLC cell of origin [56,57,58]. In vitro and in vivo studies indicated that NSCLC stem cell populations expressing the CD133 marker [59,60,61] or ALDH (aldehyde dehydrogenase) isozymes [62] show both tumorigenic and clonogenic activity [51,60,61,62]. In fact, the number of CD133+ and ALDH+ cells is higher in the cancerous NSCLC niche, rather than in normal lung tissue [51,60]. Moreover, the increase of CD133+ and ALDH+ cells was observed after cisplatin treatment, suggesting them as a drug-resistant population [51,60,61]. A higher expression of CD133 molecule occurs with the phenomenon of tumor vasculogenic mimicry [51,63], while a higher expression of ALDH isoenzymes is common in LUADs, never-smokers and females [51,64]. On the other hand, studies on mouse models indicated that the putative cell of origin for LUAD and LUSC arises from peripheral bronchial epithelial cells and tracheobronchial basal cells, respectively [56,65]. The differentiation of LUSC and LUAD from these cellular origins seems to be influenced by driver alterations; however, in both subtypes the drivers may be acquired by exposure to different carcinogenic factors [55,57,66,67,68]. It was also indicated that treatment can easily shape LUAD into LUSC, proving the high rate of plasticity of NSCLC cells [31,52,56]. This phenomenon may be associated with the fact that a high basal cell signature is observed in the clinically aggressive phenotype of LUAD [55,57,66,67,68]. Indeed, studies on in vitro and in vivo models proved that transduction of the NOTCH signalling pathway may have an effect on self-renewal of basal stem cells, resulting in increased tumorigenicity and chemoresistance [51,60,69]. Simultaneously *NOTCH* gene alterations are more frequently observed in LUAD [51,70], which might explain the phenomenon of LUAD transmission into LUSC.

## 4. General Tumor Evolution Theory

**Figure 1:**
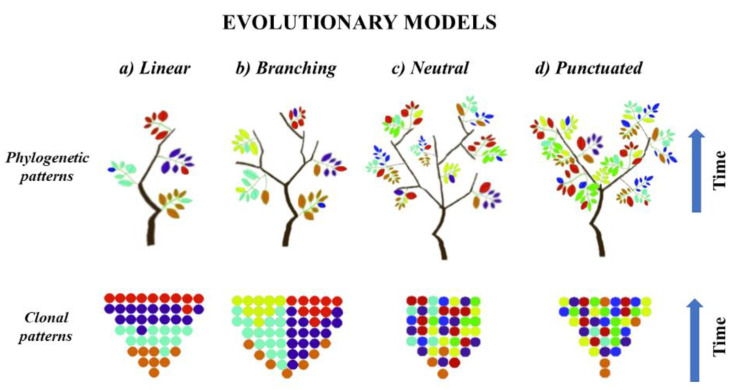
Phylogenetic and clonal patterns of tumor evolution. Color of leaves and dots indicate clones with different genotypes. (**a**) Linear model indicates the selective sweep of other clones from the phylogenetic tree by the dominant genotype. (**b**) Branching model indicates the simultaneous presence of multiple clonal selection. (**c**) Neutral model indicates the absence of selective sweep and accumulation of random genetic alterations over time. (**d**) Punctuated model indicates the absence of selective sweep and appearance of heterogeneous genotype at the early stage of tumorigenesis, without further subclonal selection.

As mentioned above, tumor evolution may be characterized by acquisition of driver mutations in cells of origin, which was assumed to be a critical step of clonal expansion and seeding of neoplasm into TME [17,71,72,73]. This process may occur in different evolution models that manifest with low or high ITH. In the linear evolution model (Figure 1a) tumor cells acquire sequential genomic changes and the clone, which contains most favourable genomic background, selectively sweeps other clones from the phylogenetic trunk and becomes dominant [5,6,20,21]. Based on this assumption, the drivers accumulate gradually in each clone, leading to the omnipresence of major drivers in the clone that seeds the local microenvironment [6,74]. On the other hand, branching, neutral and punctuated evolution models state that subclonal diversity appears at early stages of tumorigenesis, with a few dominant clones that expand to form the tumor mass [6,75]. In the branching model (Figure 1b), various clones diverge in parallel from a common ancestor and evolve, leading to multiple subclonal lineages that exist independently within the primary tumor niche, thus generating extensive ITH [5,6]. Moreover, in the branching model the aggressive subclones may achieve a clonal sweep leading to a clinically heterogeneous profile of the tumor [76]. In contrast, the neutral and punctuated evolution models (Figure 1c,d) assume extreme truncal branching without a selective sweep, but with accumulation of random alterations over time, which results in extensive ITH. However, in these two models the cancer-driving alterations are selected at the beginning of tumorigenesis and have limited impact on cancer progression [1,6,23,75]. There is little phylogenetic difference between the neutral and punctuated models. In the punctuated model, the evolution of genomic aberrations occur in a short time at the earliest stages of tumorigenesis, while in the neutral model, the fast dispersion of genomic burden occurs at a late stage [6,23,28]. Due to the fact that subclonal diversity in punctuated evolution appears at early stage of tumorigenesis, this implies limited ITH, as in linear model [6,75]. Thus, both models may be comprehensively sampled by a single biopsy, while branching and neutral evolution suggest that ITH is extensive and requires multi-sampling approaches from different spatial regions to detect all of the clinically relevant alterations in the tumor [6,23,29,44]. Most solid cancers show a single model of tumor evolution; however, it has been confirmed that models may undergo transitions over time, or multiple models may operate simultaneously for different classes of alterations [6,23]. In particular, there is an assumption that linear evolution is a common event at early stages of tumorigenesis and its further transformation into another model depends on the effect of acquired drives in the phylogenetic trunk [1,6,23,49,75]. This hybrid model suggests that most of drivers are acquired in the initial stages of tumorigenesis, and then clones expand without particular selection [23,75]. However, the type of driving alterations may dictate the type of evolutionary trajectories, confirmed by data indicating that point mutations commonly follow the branched evolution model while CNAs and chromosomal structural variants are preferably shared in the punctuated model [3,6,11,13,29,75]. This way, the subclonal alterations will be present in only a subset of cells that affect prognosis and susceptibility to therapy [25]. In fact, tumorigenesis may also be driven by a subclone that does not harbour important evolutionary alterations but instead stimulates the growth of all tumor cells, maintaining clonal diversity [77]. This scenario may result in a misleading assumption that the absence of a dominant clone in a tumor is evidence of neutral evolution [1,6,23,75]. Primary tumors of NSCLC grow in neutral or branched evolution models, which are the most accepted ways of seeding in the primary lung TME; however, the distant spreading of NSCLC may be driven by various models [6,16,25,27,53,78].

## 5. Clinical Implication of Driver Alterations

According to the cell of origin theory, the occurrence of driver mutations should be considered as a key predisposition leading to development of NSCLC [55,57,66,67,68]. In this concept, the genetic background of NSCLC sharpens through a long period of latency when the niche of NSCLC stem cells may be exposed to exogenous mutagenic factors [56,57]. Under this condition, a selected genetic landscape may be preserved through CSCs self-renewal, clonal proliferation and differentiation, as well as metastases formation [9,58,79,80,81]. Tumor evolution, driven by genetic alterations, is especially associated with development of LUAD in never-smokers [33,35,38,82]. These patients harbour mainly alterations in tumor suppressor genes and genes that encode growth factor receptors with tyrosine kinase activity [38,82]. However, the evidence suggests that the key initial events of tumorigenesis in smoking related LUADs and LUSCs are mutations in *P53*, *CDKN2A*, *PTEN*, *PIK3CA*, *KEAP1*, *NOTCH1* and *RB1* suppressors [28,35,37,38]. This genetic similarity between LUAD and LUSC in smokers may also explain the phenomenon of pathologic transitions between these subtypes induced by the treatment [55,56,79]. On the other hand, many driver alterations in NSCLC are considered ‘druggable’, and the development of agents targeting them has revolutionized the management of NSCLC treatment [30,48,83]. In particular, in genetically selected LUAD patients, molecularly targeted therapies provide significant superiority over standard platinum-based chemotherapy, and there are attempts to obtain similar results in LUSC [30,84]. In Table 2 we summarized the common driver alterations associated with LUAD and LUSC, indicating their frequency in TCGA (The Cancer Genome Atlas) database, as well as matching them with a potential clinical trial.

The frequency of listed alterations was evaluated in the cBioPortal [85,86] database in eight pre-selected published studies that included 2878 primary tumors of NSCLC [25,35,82,87,88,89,90,91]. Due to the fact that it was a pooled analysis, the genes’ incidences may vary in different populations, as well as in other publicly available data. The clinical status of available targeted therapies (registered by EMA (European Medicines Agency) and/or FDA (U.S Food and Drug Administration) and active clinical trials, recruiting or open, but not-recruiting yet, was collected from linalTrials.gov database [92].

## 6. Metastatic NSCLC Cells

Due to the slow nature of cancer progression and its latent spread, the process of distant metastases seeding remains incompletely understood [16,17]. Indeed, NSCLC dissemination is an extremely complex process including involvement of the tumor microenvironment (TME) [71,93,94], CSCs properties, systemic biology of the organism, as well as genetic signatures [53,72]. Elucidation of this processes is clinically important as metastatic disease causes the majority of cancer deaths [17,19,32]. It was shown that genetic background may play an important role in supporting the movement of cancer cells to the specific organs where they form metastases [12,78,95]. Primary tumors accumulate most of the alterations vital to metastatic spread, thus NSCLC dissemination may arise from metastatic stem cells (MetSCs) [53,54,72] that phylogenetically evolve from the CSCs through tumor progression, sharing with them many genetic similarities [72,80,96]. On the other hand, MetSCs may appear de novo as a result of competition for a niche between CSCs and non-CSCs that demonstrate divergent genetic patterns [53,80,93]. Several in vitro studies have shown that cellular cross-talk may reduce cell-cell adhesion, enabling cells to separate from each other and invade through the basement membrane [12,97,98]. This process may be driven by signals from the TME; however, the epithelial-to-mesenchymal transition (EMT) is considered the hallmark of metastatic spread. EMT phenomenon posits that cancer cells partially or completely lose their epithelial properties, detach and travel as single cells, and form clonal or subclonal metastases [12,78,98,99,100,101]. However, studies indicate that MetSCs, which have undergone EMT, still exhibit a CSCs phenotype [73,99], suggesting that genes involved in EMT may be key targets in eradicating the CSCs population [95,98,99]. This might lead to reduction of cancer dissemination [73].

Microarrays, quantitative-PCR (qPCR) and RNA sequencing approaches have been widely used to study the patterns of EMT gene expression in various cancer cells [102,103,104,105,106,107]. It was observed that EMT changes are often related to downregulation of epithelial proteins (e.g., E-cadherin), as well as to upregulation of mesenchymal proteins (e.g., N-cadherin and Vimentin), which are considered as hallmarks promoting dissemination by migration and invasion [12,99]. It was shown that variations in EMT-associated gene expression depend on plasticity of primary and metastatic niches [108]. Moreover, smoking can induce the EMT process in NSCLC [31,52,98,109]. Simultaneously, smoking-related NSCLC exhibits lower expression of E-cadherin and higher expression of Vimentin [98,110]. Induction of EMT confers resistance of NSCLC cells to EGFR-tyrosine kinase inhibitors; however, it may be re-sensitized by enforced inhibition of other pathways e.g., the Hedgehog pathway [73]. A list of 279 EMT genes putatively involved in NSCLC evolution is summarized in Table 3 according to the EMTome database [111].

## 7. Clonality of Metastases

As we indicated above, tumor evolution is a multistep process that involves interaction between cancer cells and their surrounding microenvironment [16,112]. However, especially during dissemination, the primary tumor cells need to gain specific features enabling them to move out of the primary niche, as well as to survive and proliferate at a foreign distant site [12,17,76,113]. Among many cancerous cells, only a small number of subclones have the potential to successfully navigate the colonization of secondary organs; therefore dissemination is considered an evolutionary inefficient process [17,112,114]. Systemic spread can start at early stages of tumorigenesis, even several years before diagnosis of the primary tumor [115]; thus, both primary and metastatic sites evolve simultaneously sharping the clonally unique landscapes [116]. Based on the clonal relationships between a primary tumor and its metastases, dissemination may happen in a monoclonal or polyclonal model [7,117]. The monoclonal trajectory assumes that the most advanced primary clone seeds the metastases linearly at a late stage of tumorigenesis, resulting in minimal genetic divergence between the primary tumor and its metastases [7,10,50,117]. On the other hand, in the polyclonal hypothesis, multiple distinct clones seed the metastatic environment at an early stage of tumorigenesis cooperatively or independently [50,117]; then both clones evolve in parallel, affected by different external factors. As a result of polyclonal evolution, a high level of genetic divergence between the primary and the metastatic lesions is observed [7,10,15,116]. Depending on which model the primary tumor evolves from, the metastases can be clonally homogeneous or heterogeneous [12,45,50,52]. The monoclonal model has been suggested as a predominant mode of metastasis, regardless of primary cancer type or metastatic site [117,118]. This progression mode was observed in LUAD mouse models [7,56]. However, high subclonality of primary tumors of NSCLC leads to multiple, distinct metastases, suggesting polyclonal evolution [4,50,77]. Moreover, polyclonal seeding is common both for untreated metastatic lymph nodes and distant metastases, whereas treated distant metastases are monoclonal [115,119]. These observations suggest that treatment of primary lesions has a strong selection pressure for tumor evolution. Thus, distinction of both models allows understanding of the subclonal complexity of the primary tumor and its relationship between subclones present at the metastatic sites [4,7,43,115].

It is postulated that all cancer cells carry a lineage of specific alterations that may play a role in their evolution [95]. In many somatic CNAs or SNVs (single nucleotide variations) minor subclones in the primary tumors of NSCLC may become fully clonal in distant metastasis [13,41]. In this scenario, the metastatic ancestor is already present in a minor population of primary tumor cells and may become dominant through a bottle-neck event driven by selective clonal growth or selective treatment pressure [35,45,46,47]. It was indicated that subclonal driver heterogeneity is high between untreated metastases and primary tumors [115], while in treated metastases the proportion of pre-selected clonal drivers increased dramatically [76,117]. In particular, the treatment induced a significantly higher number of actionable mutations in genes encoding proteins involved in PI3K-AKT and HER/ErbB pathways [30,76,120]. As part of treatment pressure, the origin of primary and metastatic niches affects the selection of the clonal alteration repertoire in metastatic lesions [13,119,120]. A pan-cancer evolution study suggested that clonal dissemination exhibits organ specificity, and NSCLC shows a common spreading pattern to lymph nodes, liver, bones and brain; however, the mechanisms underlying colonization tropism remain unclear [22,81,115,116,121]. Moreover, the mutations in clonal tumorigenic drivers e.g., in *TP53*, *EGFR*, *KRAS*, and *KEAP1* genes were associated with higher risk of distant metastasis [59,77,116,119].

In general NSCLC prefers late dissemination to distant organs, while colonization of local lymph nodes happens relatively early when the primary tumor is small [114,119]. Therefore, lymph node metastases are often detected simultaneously with primary tumor [17,115,116]. This confirms that lymph node metastases are seeded a long before clinical detection and, at that moment, they do not contribute to migration to other sites; however, they evolve in parallel with primary tumors sharping the genetic divergence [116,122]. Primary NSCLC, and its corresponding lymph node metastases, present high concordance in clonal alterations, indicating that local metastases may arise from the major clone of primary tumor [114,123]. Moreover, the *NPIPA1* gene mutation and *NKX2/1* gene amplification are evident drivers of lymph nodes metastases [116]. Due to a common ancestor, primary NSCLC and its lymph node metastases should be susceptible to the same treatment regimen. However, the genetic heterogeneity between both lesions seems to be the main reason for recurrence after surgical resection [123,124]. Lymph nodes metastases may be considered a reservoir for distant spreading of NSCLC; however, distant metastases that were seeded from the primary tumor or metastatic lymph nodes indicated divergent evolutionary trajectories [114,116,125]. Moreover, it was confirmed that the most distant metastases of NSCLC are commonly seeded by the primary tumors [10,13,16,17].

Genetic comparisons of primary and metastatic NSCLC lesions have revealed that metastases are frequently characterized by a higher burden of somatic SNVs [13,126] and CNAs [13,76,114]. This may be proof that only cells with high alterations burden are able to survive the immune attack during dissemination and become metastatic clones, which after seeding further evolve and sharpen genetic divergence [9,13,29,117]. NSCLC evolutionary studies indicated that up to 50% of clinically relevant alterations were identified privately in metastatic samples, while they were not significant in primary or other distant metastatic lesions [8,9,118,125,126]. In particular, studies have shown that clones seeding the brain niche may be clonally distinct from clones seeding other sites [13,118]. Moreover, brain-seeding clones acquire early clonal divergence from primary clones with increased numbers of distinct alterations in PI3K, EGFR, ErbB2 (HER2), ALK, Wnt/b-catenin and EMT signaling pathways, which seems to be crucial in spreading of cancers to the brain [13,54,120,127]. On the other hand, mutations of major driver genes, including *EGFR*, *KRAS*, *TP53*, and *ALK* are highly concordant between primary NSCLC and matched brain metastatic lesions, suggesting that early clonal genomic events during carcinogenesis of NSCLC may be involved in dissemination to the brain [116,118,128]. However, amplifications of *MYC*, *YAP1*, *RICTOR* and *MMP13* genes and deletions of *CDKN2A/B* genes [128], as well as mutations in less common cancer genes [116], are considered putative drivers of brain metastases. In one of our recent studies, we indicated that amplifications of potentially targetable genes such as *CDK12*, *DDR2*, *ERBB2*, and *NTRK1* may also drive NSCLC spread to brain (results under review [129]). Despite the fact that several studies have undertaken the identification of metastasis drivers [13,121,126,128], further application of multiregional NGS and single-cell sequencing approaches may reveal this process and explain the complexity of seeding routes [29,49,50,130]. We summarize genetic alterations that significantly imply the potential importance to drive NSCLC tumorigenesis or its metastasizing in Table 4.

The alteration frequency was reported in 2878 primary tumors of NSCLC from eight pre-selected published studies [25,35,82,87,88,89,90,91]. The incidence was evaluated in cBioPortal [85,86] database. We did the pooled analysis of a gene’s indicated in cBioPortal datebase without differentiation of demographic factors. Therefore the incidence may vary in different populations, as well as in other or single publicly available data.

## 8. Conclusions

For many decades, knowledge about NSCLC evolution was limited and affected the therapeutic strategies. Then, the beginning of NGS era provided a wide array of data about the genetic background of NSCLC. This has allowed application of molecularly targeted therapies and immunotherapies that have revolutionized the management of NSCLC treatment. Moreover, the application of NGS-based techniques to studies on ITH has provided new knowledge about clonality contribution to both primary seeding and distant dissemination. Finally, the application of multiregional sequencing has revealed a high level of ITH within primary and corresponding metastatic sites, which implies both evolutionary and clinical challenges. Despite this wide progress, current treatments strategies mostly rely on the theory that metastases are genetically similar to the primary tumor from which they arise. This is due to the fact that, until now, common targetable alterations that drive dissemination have not been identified. Moreover, there is no clear explanation about the association between cellular origin, metastatic origin and genetic clonality. The reason for this may be the observation that behind the genetic scene epigenetic and metabolic factors play a very important role in tumor evolution. This indicates a huge gap in the field that must be filled to reveal the background of all theoretical aspects of NSCLC evolution. More studies on ITH, especially in multiple geographically distinct areas, of the same tumor and its metastases taken from the same patient at different time points, are needed to understand the complexity of evolutionary trajectories of NSCLC. Single-cell sequencing [130], spatial transcriptomic [131] and studies on CSC spheroids [132,133] may bring us an important step closer to understanding the evolutionary relationship between primary and metastatic lesions. Moreover, revealing whether primary tumor cells innately contain the capability to metastasize, or they acquire it within evolution, will have high clinical significance.

## Figures and Tables

**Table 1:** The summary of clonality character of the commonly altered genes in primary tumors of NSCLC reported by literature data.

Type of Driver Alterations		Gene	Clonality Type
Mutational level	SNVs, substitutions, small indels, genes rearrangements/fusions	*TP53*	clonal/subclonal
		*KRAS*	clonal/subclonal
		*EGFR*	clonal
		*KEAP1*	clonal
		*KMT2C*	clonal
		*KMT2D*	clonal
		*NF1*	subclonal
		*PIK3CA*	subclonal
		*COL5A2*	clonal
		*ATM*	subclonal
		*ARID1A*	clonal
		*BRAF*	clonal
		*NOTCH1*	clonal/subclonal
		*ALK*	clonal
		*PTEN*	clonal
		*MET*	clonal
CNAs level	Amplifications	*TRIO*	subclonal
		*SOX*	clonal
		*PIK3CA*	clonal
		*TERT*	clonal/subclonal
		*NKX2-1*	subclonal
		*TP63*	clonal
		*MUC1*	subclonal
		*MYC*	subclonal
		*RICTOR*	subclonal
		*FGFR1*	clonal
		*CCND1*	subclonal
		*CDK4*	subclonal
	Deletions	*CDKN2A*	subclonal
		*CDKN2B*	subclonal
		*DMRTA1*	subclonal
		*STC1*	subclonal

**Table 2:** Summary of driver alterations, the presence of which is associated with development of LUAD and LUSC.

NSCLC Subtype	Driver Gene	% TCGA Frequency	Targeted Drug	Status (Registered/Open Trial)
non-smoking related (LUAD)	*EGFR*	16.6%	erlotinib, gefitinib, afatinib, dacomitinib, osimertinib	registered
	*KEAP1*	15.2%	telaglenastat	NCT04265534
			sotorasib	NCT05054725
	*STK11*	13.2%	talazoparib	NCT04265534
			sotorasib	NCT04933695
	*BRAF*	5.6%	dabrafenib + trametinib	registered
			encorafenib + binimetinib	NCT03915951
	*ALK-EML4* (fusion)	2.9/2.5%	crizotinib, alectinib, ceritinib, brigatinib, lorlatinib	registered
	*ROS1* (fusion)	1.3%	crizotinib, entrectinib	registered
			repotrectinib	NCT03093116
	*MET*	3.6%	crizotinib	breakthrough therapy NCT04084717
			tepotinib	registered NCT04739358 NCT04647838
			capmatinib	registered NCT04427072 NCT03693339
			capmatinib + spartalizumab	NCT04323436
			cabozantinib	NCT03911193
			ningetinib	NCT04992858
	*RET* (fusion)	1.5%	cabozantinib	NCT01639508 NCT04131543
			selpercatinib	registered NCT04194944 NCT04268550
			entrectinib	NCT04302025
			pralsetinib	registered NCT03037385 NCT04222972
			anlotinib	NCT04073537
Smoking related (LUAD + LUSC)	*TP53*	60.1%	ALRN-6924	NCT04022876
			tedopi	NCT04884282
	*KRAS*	24%	binimetinib + palbociclib	NCT03170206
			sotorasib	registered NCT05118854
			JDQ443	NCT05132075
			adagrasib	breakthrough therapy NCT04685135
			sotorasib + RMC-4630	NCT05054725
	*PIK3CA*	8.3%	serabelisib + canagliflozin	NCT04073680
			TPST-1495	NCT04344795
	*BRCA1/2*	7.5%	rucaparib	NCT03845296
			olaparib + cediranib + durvalumab	NCT02484404
			niraparib + pembrolizumab	NCT04475939
	*CDKN2A*	7.3%	erlotinib + trastuzumab	NCT04591431
	*RB1*	5.6%	IBI188 + GM-CSF	NCT04861948
	*NOTCH1*	5%	tegavivint + osimertinib	NCT04780568
	*PTEN*	4.2%	elemene + 1st EGFR-TKIs generation	NCT04401059

**Table 3:** Summary of 279 EMT genes with up-streaming or down-streaming associated with NSCLC evolution.

*ACTA1* ^ 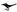 ^	*CDC42* ^ 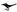 ^	ELMO3 *	GRHL2 *	LTBP4 *	*PARP1* ^ 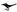 ^	SH3YL1 *	*TIMP1* ^ 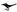 ^
ACTN1 *	CDH1 *^” ^ 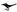 ^	EPB41L5 *	*GSC* ^ 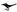 ^	MAF *	*PDGFB* ^ 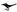 ^	SHANK3 *	TIMP2 *
ADGRF4 *	*CDH2 ”^* ^ 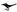 ^	EPHA1 *	*GSK3B* ^ 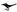 ^	MAL2 *	PDLIM7 *	SHROOM3 *	*TJP1 ”*
AFAP1L2 *	CDH3 *	EPHB2 *	HNMT *	*MAP1B* ^ 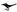 ^	PEA15 *	*SIP1* ^ 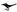 ^	TJP3 *
*AHNAK* ^ 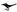 ^	CDS1 *	EPN3 *	*HRG* ^ 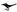 ^	MAPK13 *	PIK3CD *	SKIL *	TMC4 *
*AKT1* ^ 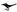 ^	CERCAM *	EPPK1 *	*IGFBP4* ^ 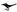 ^	MAPRE2 *	PLAUR *	*SMAD2* ^ 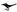 ^	TMC5 *
*ANGPTL4* ^ 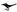 ^	CHST3 *	ERBB3 * ^ 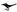 ^	*IGFBP5* ^ 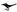 ^	MBOAT2 *	PLEK2 * ^ 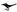 ^	*SMAD3* ^ 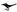 ^	*TMEFF1* ^ 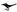 ^
ANKLE2 *	*CLDN12* ^ 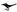 ^	*ESR1* ^ 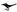 ^	IL11 *	METRNL *	*PLG* ^ 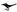 ^	SMAD7 *	TMEM125 *
ANKRD22 *	*CLDN23* ^ 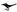 ^	EVPL *	*IL1RN* ^ 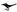 ^	*MITF* ^ 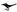 ^	PMEPA1 *	SNAI1 *^” ^ 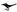 ^	*TMEM132A* ^ 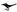 ^
ANTXR2 *	*CLDN3* ^ 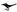 ^	EXOC6 *	*ILK* ^ 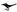 ^	MLXIP *	PPARG *	*SNAI2 ^”* ^ 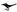 ^	TMEM30B *
AP1M2 *	CLDN4 * ^ 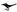 ^	*EZH1* ^ 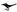 ^	INADL * ^ 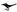 ^	*MMP10* ^ 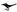 ^	PPP1R13L *	*SNAI3* ^ 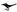 ^	TMEM45B *
ARHGEF18 *	CLDN7 * ^ 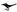 ^	*EZH2* ^ 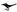 ^	*INHBA* ^ 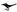 ^	MMP2 ”* ^ 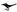 ^	PPP1R18 *	*SOX10 ”*	TNFRSF21 *
ARHGEF40 *	CMTM3 *	F11R * ^ 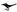 ^	*ITBG6 ”*	*MMP3 ”* ^ 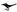 ^	*PPPDE2* ^ 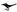 ^	*SOX11* ^ 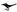 ^	TP53I3 *
AXL *	COL1A1 *	*FGF1* ^ 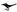 ^	ITGA5 * ^ 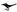 ^	*MMP9 ”* ^ 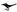 ^	PRR5 *	*SPARC* ^ 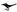 ^	TPM1 *
*BCL2* ^ 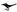 ^	*COL1A2* ^ 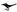 ^	*FGF5* ^ 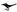 ^	*ITGAV* ^ 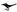 ^	MPP7 *	PRSS22 *	SPINT2 *	TRIO *
*BCL9* ^ 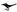 ^	*COL3A1* ^ 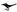 ^	*FGFBP1* ^ 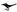 ^	*ITGB1* ^ 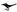 ^	MPZL2 *	PRSS8 *	*SPP1* ^ 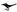 ^	TRMT10A *
BEAN1 *	*COL5A1* ^ 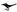 ^	FGFR1 *	*ITGB3* ^ 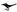 ^	*MSN* ^ 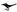 ^	*PTK2* ^ 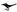 ^	SSH3 *	*TSPAN13* ^ 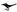 ^
BICDL1 *	COL7A1 *	*FHL1* ^ 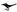 ^	ITGB6 *	*MST1R* ^ 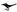 ^	*PTP4A1* ^ 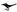 ^	ST14 *	TSPAN2 *
*BIRC3* ^ 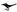 ^	CRB3 * ^ 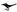 ^	FLNA * ^ 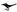 ^	*JAG1* ^ 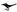 ^	*MTA3* ^ 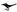 ^	PTRF *	STAP2 *	*TWIS1 ”*
BMP1 * ^ 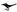 ^	*CTGF* ^ 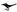 ^	*FN1 ”* ^ 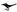 ^	JUNB *	MTAC2D1 *	PXDC1 *	*STAT3* ^ 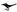 ^	*TWIST1* ^ 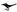 ^
*BMP7* ^ 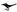 ^	CTNNA1 *	*FOXC2* ^ 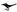 ^	JUP *	MUC1 *	*PXN* ^ 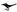 ^	*STEAP1* ^ 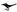 ^	VCAN * ^ 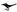 ^
BSPRY *	*CTNNB1* ^ 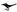 ^	*FOXC2 ”*	KCTD11 *	MUC5AC *	PXN-AS1 *	TACSTD1 *	VIM *^” ^ 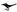 ^
C1ofr116 *	*DACT1* ^ 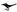 ^	FRMD6 *	KLC3 *	MUC5B *	RAB25 *	TACSTD2 *	*VPS13A* ^ 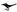 ^
C1ofr171 *	DBN1 *	FXYD3 *	KLF7 *	NAV1 *	*RAC1* ^ 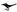 ^	TBC1D30 *	*WEE1* ^ 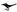 ^
C3orf21 *	*DCN* ^ 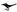 ^	*FZD7* ^ 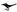 ^	*KRT14* ^ 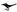 ^	NCOR2 *	RBM35A *	*TCF3* ^ 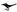 ^	*WNT11* ^ 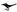 ^
*CALCR* ^ 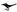 ^	DEPTOR *	*GADD45A* ^ 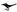 ^	*KRT7* ^ 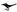 ^	NF1 *	RBPMS *	*TCF4* ^ 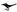 ^	*WNT2B* ^ 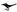 ^
CALD1 * ^ 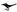 ^	*DHFR* ^ 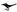 ^	GADD45B * ^ 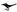 ^	KRTCAP3 *	NKAIN4 *	*RGS2* ^ 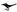 ^	*TFPI* ^ 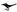 ^	*WNT5A* ^ 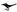 ^
*CAMK2N1* ^ 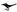 ^	*DLG1* ^ 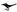 ^	GALNT2 *	KTR19 *	*NLK* ^ 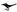 ^	RHOD *	*TFPI2* ^ 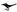 ^	*WNT5B* ^ 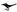 ^
CARD6 *	*DSC2* ^ 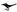 ^	GALNT3 *	*LAMB3* ^ 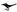 ^	*NODAL* ^ 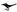 ^	S100A14 *	TGFB1 * ^ 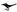 ^	WNT7A *
*CASP3* ^ 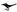 ^	DSP *” ^ 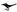 ^	GALNT5 *	LAMC2 *	*NOTCH1* ^ 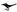 ^	SAMD4A *	TGFB1I1 *	*WT1* ^ 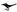 ^
*CAV2* ^ 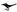 ^	EEPD1 *	*GNC ”*	LIX1L *	*NPPB* ^ 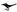 ^	*SCARB2* ^ 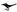 ^	*TGFB2* ^ 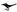 ^	*YWHAG* ^ 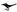 ^
*CCNB2* ^ 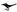 ^	*EGF* ^ 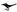 ^	*GNG11* ^ 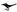 ^	*LOXL2* ^ 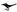 ^	*NRG1* ^ 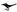 ^	SCNN1A *	*TGFB3* ^ 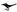 ^	ZEB1 *^ ^ 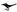 ^
*CCND1* ^ 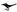 ^	*EGFR* ^ 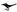 ^	GPR110 *	LRRC54 *	NRP1 *	*SCRIB* ^ 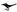 ^	TGFBR1 *	*ZEB2 ^* ^ 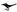 ^
*CD36* ^ 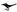 ^	EHF *	GPR56 *	LTBP1 *	*NUDT13* ^ 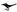 ^	SERINC2 *	*THBS1* ^ 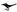 ^	ZFP36L1 *
*CD47* ^ 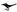 ^	*ELANE* ^ 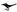 ^	GRHL1 *	LTBP3 *	*OCLN* ^ 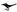 ^	SERPINE1 * ^ 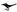 ^	THRB *	

^
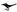
^ genes described in the literature [102]; * genes whose contribution was evaluated by microarrays [103,104,105], *^* genes whose contribution was evaluated by qPCR [106], *”* genes whose contribution was evaluated by RNAseq [107].

**Table 4:** Summary of putative drivers of metastases to lymph nodes and brain, with their frequency in primary tumors of NSCLC.

Metastatic Site	Alteration	Gene	% TCGA Frequency in Primary NSCLC
lymph nodes	SNVs	*NPIPA1*	0.3%
	CNAs (amplifications)	*NKX2/1*	8%
brain	SNVs	*KMT2C*	10.4%
		*AHNAK2*	10%
		*PDE4DIP*	7.5%
		*ANKRD36C*	4%
		*BAGE2*	0.2%
	CNAs (amplifications)	*MYC*	7.4%
		*RICTOR*	5.7%
		*DDR2*	3.6%
		*NTRK1*	3.5%
		*ERBB2*	2.5%
		*CDK12*	1.8%
		*MMP13*	1%
		*YAP1*	0.7%
	CNAs (deletions)	*CDKN2A*	15.3%
		*CDKN2B*	15%
